# It Takes Two to Tango: Development, Validation, and Personality Correlates of the Acceptance of Sugar Relationships in Older Men and Women Scale (ASR-OMWS)

**DOI:** 10.3389/fpsyg.2021.592138

**Published:** 2021-04-09

**Authors:** András Láng, Béla Birkás, András N. Zsidó, Dóra Ipolyi, Norbert Meskó

**Affiliations:** ^1^Institute of Psychology, University of Pécs, Pécs, Hungary; ^2^Department of Behavioral Sciences, Medical School, University of Pécs, Pécs, Hungary

**Keywords:** acceptance of sugar relationships, scale development, validation, personality correlates, transactional sex

## Abstract

Sugar relationships can be considered contemporary forms of transactional sex, that is, offering sexual services for material resources or other benefits. Considering the common age differences in these relationships, sugar relationships might be of relevance for older adults as well on the mating market. As a sequel to Birkás et al. (2020), in the present study, an attitude scale was developed to assess older women’s and men’s acceptance of sugar relationships. We also explored whether the acceptance of sugar relationships was associated with love styles, sociosexual orientation, sexual motivation, and certain socially aversive personality traits. In two online studies with a total number of 836 participants (*N* = 277 women and 559 men), the results showed that the Acceptance of Sugar Relationships in Older Men and Women Scale (ASR-OMWS) proved to be a reliable and conceptually valid measure of older individuals’ attitude toward sugar relationships. A more accepting attitude toward sugar relationships was found to be associated with more unrestricted sociosexuality, preference to engage in playful love relationships and more self-focused sexual motivation (study 1; *N* = 481, 167 women and 314 men), and with more pronounced Dark Triad and borderline traits (study 2; *N* = 355, 110 women and 245 men). Our findings are discussed in an evolutionary framework.

## Introduction

Sugar relationship can be described as a form of affair between a well-to-do man (sugar daddy) and less frequently woman (sugar mommy) who is willing to financially compensate (through monetary or other form of rewards) his/her young in-need partner (sugar baby; a female, or less frequently, sugar boy, a male) in return for a form of companionship they agreed upon (Nayar, 2016). This transactional form of relationship could be well understood and investigated in a model describing the variations of mating preferences referred to as the “mating market” (Pawlowski and Dunbar, 1999). As suggested by its name, the mating market describes a two-way process of finding romantic partners. Individuals on the market advertise their potentially desirable characteristics as a potential partner, whereas they also proclaim desired qualities of their potential mates (Pawlowski and Dunbar, 1999). Sugar relationships might be specific in the sense that advertised and desired partner qualities are explicitly stated and negotiated.

Within the evolutionary context, human mating strategies vary according to the individual’s reproductive potential and reproductive investment (Buss, 1994). Since women invest more in parenting (a potential outcome of mating compared with men), they show stronger preferences for partners with signs of capability of parental investment (i.e., financial stability or higher level of education; Buss, 1989; Anderson and Klofstad, 2012; Walter et al., 2020). More for men than for women, partners with physical signs of reproductive potential (youth, attractiveness) are evaluated as more desirable mates (e.g., Buss, 1989; Walter et al., 2020). However, it is important to note, that in long-term relationships, both men and women prefer physical attractiveness, but men value it more (Buss, 1989; Walter et al., 2020).

Mating preferences also vary according to the “mate value” of the individual, that is, the ability and willingness to invest resources in the relationship (i.e., quality and stability) and in raising offspring. The structure of the mating market suggests that individuals with higher mate value are rated as more desirable by potential partners; thus, they can aim for a similarly high-valued partner. Correspondingly, individuals with more desirable traits can expect higher mate value for their “offer” in the mating market (Pawlowski and Dunbar, 1999). Reproductive potential declines with age, especially for women. As a result, mating strategies might change over time, but basic mate preferences only decrease in magnitude (Buss, 1989; Li et al., 2002; Conroy Beam and Buss, 2019; Conroy-Beam et al., 2019; Walter et al., 2020). Thus, both older women and men value physical attractiveness and social status of the potential partner as less important and prefer indicators of commitment and other traits promoting to engage in a more intimate relationship (Alterovitz and Mendelsohn, 2011; Fales et al., 2016). Moreover, older individuals tend to be less selective and expect less mate value from their partners; perhaps as a trade-off due to the decrease of their mate value (lower levels of health and physical attractiveness) (Li et al., 2002; Fales et al., 2016; Seto, 2017). Accordingly, sugar relationship can be considered an alternative platform for adaptive mating preferences. Older individuals in modern societies frequently accumulate financial resources which they can possibly offer to potential partners, whereas the companionship of younger individuals can be valuable not only for intimacy but also for increasing the social prestige of the older partner.

Personality traits are keys to advertise and to identify desirable characteristics on the mating market (e.g., Birkás et al., 2018). With age, mating preferences shift from physical traits to more personality-related characteristics (Alterovitz and Mendelsohn, 2011; Fales et al., 2016). Thus, it is plausible to suggest that personality traits and individual attitudes play a central role in relationship choices and might be important factors in forming sugar relationships, but in different ways for younger and older counterparts. Birkás et al. (2020) recently published their research report about a questionnaire that was developed to measure the accepting attitude of young women and men toward a sugar relationship. However, sugar relationships are dyadic and asymmetric (e.g., Nayar, 2016; Hoss and Blokland, 2018). The previously mentioned measure only taps the mindset of younger partners, who provide companionship and/or sexual relationship for resources. This paper presents the development of a supplementary questionnaire to measure the acceptance of sugar relationships among older men and women, i.e., among those who might be willing to provide resources for (sexual) companionship.

## Aim of the Current Studies

The primary aim of the current studies was to develop and validate an instrument that allows for the measurement of attitudes toward sugar relationships in older adults. Thus, these studies are a sequel to the studies in Birkás et al. (2020). Using the same instruments as in Birkás et al. (2020) also allowed us to compare the pattern of correlations in the two samples as a function of age. Thus, these studies also add to our knowledge the nomological network of the attitude toward sugar relationships in two different age groups. Furthermore, our study also aimed to provide some additional support for the mating market model and the relevance of personality-related factors in forming mating strategies and preferences when bargaining for a partner. As basic mate preferences only decrease in magnitude with age (Buss, 1989; Li et al., 2002; Conroy Beam and Buss, 2019; Conroy-Beam et al., 2019; Walter et al., 2020), in this older sample, we expected the same pattern of correlations as reported in Birkás et al. (2020).

## Method

### Sample and Procedure

We used two separate samples in this study. The first sample completed the Acceptance of Sugar Relationships in Older Men and Women Scale (ASR-OMWS), Love Attitudes Scale, Short Form (LAS-SF), Sociosexual Orientation Inventory, Revised (SOI-R), and Reasons for Having Sex Questionnaire, Hungarian Short Form (YSEX?-HSF) and consisted of 481 Hungarian participants (314 males), aged 40–69 years (M = 47.8, SD = 6.46). The second sample completed the ASR-OMWS, Borderline Personality Inventory (BPI), and Short Dark Triad (SD3) and consisted of 355 Hungarian participants (245 males), aged 40–71 years (M = 48.7, SD = 7.43). (See complete demographic data for both samples in Table 1).

**TABLE 1:** Demographic data of the two samples of the study.

Demographics	First sample	Second sample
		
	*N* = 481	*N* = 355
**Relationship status**		
Currently single	12.1%	15.2%
Has casual relationships but no permanent partner	6.2%	5.9%
Is in a committed relationship/married but does not live with the partner	15.4%	12.7%
Is in a committed relationship/married and lives with the partner	66.3%	66.2%
**Registered website user**		
Registered at a dating site	13.7%	13.8%
Registered at a site designated to arrange sugar relationships	4.4%	2.5%
Registered at both types of sites	5.0%	5.9%
Not registered at either type of sites	76.9%	77.7%
**Currently involved in a sugar relationship**	**8.3%**	
Lifetime sexual partners		
0	0	0
1	3.7%	1.4%
2	3.5%	5.9%
3	3.3%	6.8%
4	4.4%	4.5%
5–6	11.6%	5.6%
7–9	12.1%	15.8%
10–19	22.5%	40.8%
20 or more	38.9%	19.2%
**Place of residence**		
Small village	2.9%	5.6%
Large village	2.1%	3.1%
Small/medium-sized town	13.1%	13.5%
Municipal town/city	19.5%	20.6%
Capital city and its agglomeration	62.4%	57.2%

We used separate samples for the love, sociosexuality, sexual motivation (first sample), and personality trait (second sample) scales as participants filled out these scales as part of various other, larger studies. Data were collected online. The survey was edited in Google Forms. The link to the survey was disseminated *via* Facebook and *via* one of the most popular and influential Hungarian Internet portals to time, Index^1^. All participants gave informed consent, and none of them was rewarded for participation. The research plan received ethical approval from the Hungarian United Ethical Review Committee for Research in Psychology (Ref. No. 2018/115 and Ref. No. 2019/51).

### Item Generation and Selection

Since the items were identical in ASR-YWMS (Birkás et al., 2020) and ASR-OMWS with the exception of the subject of the sentences, we used reformulated items from ASR-YMWS (Birkás et al., 2020). For further information about item generation, see Birkás et al. (2020) (see the questionnaire in Appendix 1). To test the psychometric feasibility of the one-factor short form of the questionnaire, we pooled the sample of the two studies (*N* = 836, 277 women and 559 men, age range: 40–71, M = 48.2, SD = 6.90) and conducted a confirmatory factor analysis (CFA) with the robust weighted least squares with mean and variance adjustment (WLSMV) estimator. The single-factor CFA (CFI = 0.992; TLI = 0.984; RMSEA = 0.055 [90% CI = 0.028–0.083]; SRMR = 0.017) showed an excellent fit (based on the cutoffs proposed by Browne and Cudeck, 1992 and Hu and Bentler, 1998). An exploratory principle component analysis supported the unidimensional nature of the scale. Component loadings on the single component ranged from 0.697 to 0.857 for the five items. The single component explained 80.71% of the five items’ total variance.

### Instruments

#### Acceptance of Sugar Relationships in Older Men and Women Scale

The ASR-OMWS is the scale whose development, reliability analysis, and validation were the objectives of the present study. The item generation procedure was as presented previously (for the questionnaire, see Appendix 1), while the psychometric properties of the scale are discussed below. The scale contains five items. Cronbach’s α values were 0.95 and 0.93 for the first and second samples, respectively.

#### Love Attitudes Scale, Short Form

The LAS-SF (Hendrick et al., 1998; adapted to Hungarian by Meskó et al., 2021) contains 24 items that compose the following six subscales: Eros (erotic, romantic, passionate love style), Ludus (game-playing love style), Storge (affectionate, friendship-oriented love style), Pragma (rational, shopping-list love style), Mania (possessive, dependent love style), and Agape (selfless love style). Each subscale has four items, and respondents indicate the extent to which each item applies to them on a five-point Likert scale ranging from 1 (strongly disagree) to 5 (strongly agree). Thus, higher scores reflect stronger identifications with specific love styles. Cronbach’s α values for the six subscales were as follows: 0.83, 0.75, 0.85, 0.66, 0.73, and 0.84 for Eros, Ludus, Storge, Pragma, Mania, and Agape, respectively.

#### Sociosexual Orientation Inventory, Revised

The SOI-R (Penke and Asendorpf, 2008; adapted to Hungarian by Meskó et al., 2014) contains nine items assessing one’s willingness to engage in uncommitted sexual encounters. The items compose three subscales measuring the three components of behavior, attitude, and desire. Responses are given on nine-point rating scales (scale anchors vary across items). Higher scores on each subscale indicate more unrestricted sociosexuality in terms of behavior, attitude, and desire. Cronbach’s α values for the three subscales and the overall scale were as follows: 0.78, 0.82, 0.91, and 0.87 for behavior, attitude, and desire subscales and for the total score, respectively.

#### Reasons for Having Sex Questionnaire, Hungarian Short Form

The YSEX-HSF (Meskó et al., unpublished data) is a self-report instrument assessing sexual motivation. The scale comprises 73 items, which compose the following three subscales: Personal goal attainment, Relational reasons, and Sex as coping. Each item is rated on a five-point scale offering the following options: 1 = “None of my sexual experiences”; 2 = “Few (…)”; 3 = “Some (…)”; 4 = “Many (…)”; and 5 = “All of my sexual experiences.” Thus, higher scores reflect higher levels of the measured sexual motive. Cronbach’s α values were as follows: 0.91, 0.91, and 0.92 for personal goal attainment, relational reasons, and sex as coping, respectively.

#### Borderline Personality Inventory

The BPI (Leichsenring, 1999) is a 53-item self-report measure of borderline personality organization (BPO). Since the non-clinical sample of study 2 was expected to show relatively mild features of BPO, the original “yes-no” response format of the BPI was replaced with four-point rating scales (ranging from “never” to “always”) more sensitive to subclinical intensity. Thus, BPI was used in a Likert scale format (for a previous application of this procedure, see Láng, 2015). The BPI measures four aspects of BPO: identity diffusion, fear of fusion, primitive defense mechanisms, and impaired reality testing. In this study, the total BPI score was used. BPI showed high internal consistency (Cronbach’s α = 0.90) as a unidimensional scale.

#### Short Dark Triad

The SD3 (Jones and Paulhus, 2014) is a 27-item self-report instrument. Its three subscales measure three socially aversive personality traits: Machiavellianism (e.g., “Generally speaking, people won’t work hard unless they have to”), subclinical narcissism (e.g., “Many group activities tend to be dull without me”), and subclinical psychopathy (e.g., “It’s true that I can be nasty”). Each subscale consists of nine items rated on a five-point Likert scale. Cronbach’s α values were as follows: 0.79, 0.71, and 0.72 for Machiavellianism, Narcissism, and Psychopathy, respectively.

## Results

Relationships between acceptance of sugar relationships and sexual motives, sociosexuality, and love styles were tested with Pearson’s correlations. Results of these analyses along with the means and standard deviations of the variables are shown in Table 2. Acceptance of sugar relationships showed significant positive associations with all three sexual motives with all correlations being low to moderate in strength. This means that individuals with a more positive attitude toward sugar relationships tended to have sex out of self-focused reasons, and they also tended to use sex as a means of coping with distress or relational problems. Regarding sociosexuality, all subscales and the total score showed significant positive associations with the acceptance of sugar relationships. All correlations were moderate in strength. Accordingly, individuals with a more positive attitude toward sugar relationships were less restricted in sociosexual orientation and more willing to engage in sexual relationships without commitment. Concerning love styles, the acceptance of sugar relationships was significantly associated with Eros (negative correlation with negligible strength) and Ludus (weak positive correlation) love styles. Acceptance of sugar relationships was unrelated to any of the Storge, Pragma, Mania, and Agape love styles. This means that individuals with a more positive attitude toward sugar relationships tended to see love as a source of pure pleasure without striving for exclusivity in their love relationships.

**TABLE 2:** Pearson’s correlations between acceptance of sugar relationships and sexual motivation, sociosexuality, and love attitudes (sample 1).

	Correlation with ASR-YWMS (Birkás et al., 2020)	ASR-OMWS (current study)	Fisher’s *r*-to-*Z* transformation	
		
	*N* = 319	*N* = 481		
			
	*r*	*r*	*Z*	*p*
**YSEX?-HSF**				
Personal goal attainment	0.457**	0.482**	–0.44	0.660
Relational reasons	–0.029	0.225**	–3.56	<0.001
Sex as coping	0.358**	0.220**	2.08	0.038
**LAS-SF**				
Eros	−0.267**	−0.193**	–1.08	0.280
Ludus	0.543**	0.540**	0.06	0.952
Storge	–0.016	0.005	–0.29	0.772
Pragma	–0.010	–0.002	–0.11	0.912
Mania	0.116	0.078	0.53	0.596
Agape	−0.154*	–0.034	–1.67	0.095
**SOI-R**				
Behavior	0.438**	0.418**	0.34	0.734
Attitude	0.396**	0.465**	–1.17	0.242
Desire	0.459**	0.466**	–0.12	0.905
Total	0.529**	0.555**	–0.51	0.610

*p < 0.01 and **p < 0.001 for correlations. *ASR-OMWS*, Acceptance of Sugar Relationship in Older Men and Women Scale; *YSEX?-HSF*, Why Have Sex—Hungarian Short Form; *SOI-R*, Sociosexual Orientation Inventory Revised; *LAS-SF*, Love Attitude Scale—Short Form.

Next, we compared the strength of the correlations with that obtained in the study of Birkás et al. (2020) that tested the same associations in a young adult sample. According to the results of Fisher *r*-to-*Z* transformations (i.e., statistical analyses that test whether two correlation coefficients are significantly different from each other; Table 2), acceptance of sugar relationships correlated significantly stronger with relational reasons as sexual motives and significantly weaker with sex as coping in the current older sample. All other correlations were statistically identical in strength.

Associations between acceptance of sugar relationships and personality traits were tested with Pearson’s correlations. The results (Table 3) revealed significant positive moderate associations between the acceptance of sugar relationships and all four personality traits. Thus, participants with a more positive attitude toward sugar relationships reported more pronounced narcissistic (weak correlation), Machiavellian and psychopathic traits (moderately strong correlations), and more pronounced signs of BPO (weak correlation).

**TABLE 3:** Pearson’s correlations between acceptance of sugar relationships and personality traits (Sample 2).

	Correlation with ASR-YWMS (Birkás et al., 2020)	Correlation with ASR-OMWS (current study)	Fisher’s *r*-to-*Z* transformation	
		
	*N* = 1,733	*N* = 355		
			
	*r*	*r*	*Z*	*p*
BPI	0.267	0.211	1.02	0.308
Mach	0.351	0.459	−2.21	0.027
Psych	0.349	0.490	−2.94	0.003
Narc	0.191	0.213	−0.39	0.697

*All correlations are significant at the level of *p* < 0.001. *ASR-YWMS*, Acceptance of Sugar Relationship in Younger Women and Men Scale; *ASR-OMWS*, Acceptance of Sugar Relationship in Older Men and Women Scale; *Mach*, Machiavellianism; *Psych*, subclinical psychopathy; *Narc*, subclinical narcissism; *BPI*, Borderline Personality Inventory.*

Next, we compared the strength of the correlations to that obtained in the study of Birkás et al. (2020) that tested the same associations in a young adult sample. According to the results of Fisher *r*-to-*Z* transformations, the associations of acceptance of sugar relationships with Machiavellianism and psychopathy were significantly stronger in the current older sample. All other correlations were statistically identical in strength.

## Discussion

The present study evaluated a self-report measure regarding the acceptance of sugar relationships in older adults. Compared with the mating strategies of young adults, there is much less known about the partner preferences of individuals in older ages. Accordingly, this is the first empirical test of the correlates of attitudes toward sugar relationships in a predominantly evolutionary approach in a sample beyond young adulthood.

Moreover, the ASR-OMWS was used to test the association between personality characteristics, relational attitudes, and attitudes toward sugar relationships in older adults. More accepting individuals can be characterized not only by a more instrumental motivation toward sex but also by respecting the relational aspect of the sugar relationship.

This might suggest that intimacy could also be a product or service that is desired and valued by older individuals (Alterovitz and Mendelsohn, 2011; Fales et al., 2016). Furthermore, older adults scoring high on ASR-OMWS preferred relationships offering fun and taking advantage of partners and were less interested in romantic and emotional love (see section “Results” for love styles). More unrestricted sociosexuality was also positively associated with the acceptance of sugar relationships showing, that despite of their older age and declining mate value, individuals willing to take part in a sugar relationship are sexually more active. These love and sexual motives might play a key role in shaping the mating strategies of older adults. Elevated sexual impulses increase the focus on the physical characteristics of the potential partner and put more value on these signs. Since older adults possess rather material resources than physical appeal, sugar relationships might represent an undertakable and accessible form of affair (e.g., Jonason and Kavanagh, 2010; Allen and Desille, 2017; Træen et al., 2019).

On the personality level, acceptance of sugar relationships was positively associated with socially aversive personality traits (i.e., Dark Triad) and BPO. This suggests that to some extent, partner preferences of older adults are affected by these personality traits. As pointed out earlier, personality affects both advertised and expected partner qualities. Thus, it forms the nature of the desirable relationship. Connecting our results with previous findings regarding the mating strategies of the Dark Triad and BPO, sugar relationship appears to be endorsed by individuals preferring short-term sexual relationships with less commitment and intimacy (e.g., Jonason et al., 2009, 2019; Lavner et al., 2015; Muñoz Centifanti et al., 2016; Birkás et al., 2018).

The question may arise as to why these psychologies are so similar (i.e., openness to offer sexual companionship in exchange for resources or openness to offer resources in exchange for sex). The provision of resources by males to the sexual partner (not specifically in exchange for sex) is an adaptive behavior that plays an important role in the functioning of long-term, emotionally committed relationships, especially in the care of offspring (Buss and Schmitt, 1993, 2019; Conroy Beam and Buss, 2019; Luberti et al., 2020). Therefore, the sensitivity of females to resources in the mating context is also an adaptive trait that may have contributed to their reproductive success in the past. However, direct sexual transaction, free from commitment and mutual reproductive goals, is part of a short-term-focused mating strategy (Anderson and Klofstad, 2012; Whyte et al., 2019; Buss et al., 2020). The psychological characteristics of this strategy, which exploits herself/himself and others, are very similar from both the supply and demand sides: to get as many benefits as possible in the shortest possible time, without considering the possible long-term consequences.

Our study has some limitations as well. Perhaps, the sample is biased due to the possibility that individuals with an increased interest in sex-related topics can be overrepresented in our sample, leading to an increased openness toward sugar relationships. Moreover, the majority of the participants had no direct experience with sugar relationships, and accordingly, their attitudes might be more biased by their personality or impulses. Still, our findings are in line with the prequel to this study (Birkás et al., 2020) showing that older adults who are accepting toward sugar relationships have similar motives and personality traits as young adults who are willing to engage in transactional sex. The most prominent difference between the results obtained from the two studies (i.e., Birkás et al., 2020 and the current study) was that acceptance of sugar relationships was weakly but significantly positively associated with relational motives for having sex in the current study with older sample. Thus, a more direct comparison of age-groups and/or inclusion of behavioral measures might be a fruitful direction of future research.

## Data Availability Statement

The datasets generated for this study are available on request to the corresponding author.

## Ethics Statement

The studies involving human participants were reviewed and approved by the Hungarian United Ethical Review Committee (http://epkeb.ttk.hu/). The patients/participants provided their written informed consent to participate in this study.

## Author Contributions

NM, AL, and BB: conceptualization. NM and DI: methodology and writing (review and editing). AL and AZ: formal analysis and investigation. AL and BB: writing (original draft preparation). AL and NM: funding acquisition and resources. NM: supervision. All authors contributed to the article and approved the submitted version.

## Conflict of Interest

The authors declare that the research was conducted in the absence of any commercial or financial relationships that could be construed as a potential conflict of interest.

## Footnotes

1 https://index.hu/

## Appendix

### Acceptance of Sugar Relationship in Older Men and Women Scale (ASR-OMWS)

A sugar relationship is a transactional sexual relationship in which an older and wealthier partner (sugar daddy/mommy) provides material resources to a younger partner (sugar baby/boy) in return for her or his companionship. Partners usually meet to spend leisure time together, and sexual activity is only involved if both partners give their consent.

Please indicate the extent to which you agree with each of the below statements using the seven-point rating scales ranging from (1) “absolutely disagree” to (7) “absolutely agree.”
